# 

**Published:** 1847-10

**Authors:** 


### CONTENTS

OF NO. IV.

### ORIGINAL COMMUNICATIONS.

### ESSAYS AND CASES.

ART. I.—Notes on Medical Matters and Medical Men in Paris. By DAVID W. YANDELL, M.D., of Louisville, Ky. .........277

ART. II.—Remarks on Fistula in Ano. By WM. M. BOLING, M.D., of Montgomery, Alabama.........................301

ART. III.—Contributions to the Geology of Kentucky. By L. P. YANDELL, M.D., and BENJ. F. SHUMARD, M.D........310

### SELECTIONS FROM AMERICAN AND FOREIGN JOURNALS.

Removal of a large Scirrhous Testicle from a man while under the Influence of Nitrous Oxide Gas.........................343

Successful Surgical Operation .................................346

Poisoning by the Arsenite of Copper ...........................346

On the Use of Eupatorium Perfoliatum, Thoroughwort, Boneset...347

Alum in Pertussis .............................................348

Observations on the Term of Utero-gestation in Man and inferior Animals.......................................................349

A Pleasant Substitute for Epsom Salts as a Purgative...............350

### ORIGINAL INTELLIGENCE.

Traveling Letters from the Senior Editor....................351, 358

Silliman's Chemistry..........................................367

Latham's Clinical Medicine....................................368

Etherizat on..................................................368

University of Louisville......................................368

Books and Correspondents......................................368

